# *Fusarium* Species and Mycotoxins Contaminating Veterinary Diets for Dogs and Cats

**DOI:** 10.3390/microorganisms7010026

**Published:** 2019-01-21

**Authors:** Natalia Witaszak, Łukasz Stępień, Jan Bocianowski, Agnieszka Waśkiewicz

**Affiliations:** 1 Department of Pathogen Genetics and Plant Resistance, Institute of Plant Genetics, Polish Academy of Sciences, Strzeszyńska 34, 60-479 Poznań, Poland; nwit@igr.poznan.pl; 2 Department of Mathematical and Statistical Methods, Poznan University of Life Sciences, Wojska Polskiego 28, 60-637 Poznań, Poland; jan.bocianowski@up.poznan.pl; 3 Department of Chemistry, Poznan University of Life Sciences, Wojska Polskiego 75, 60-625 Poznań, Poland; agat@au.poznan.pl

**Keywords:** *Fusarium*, ergosterol, fumonisin B_1_, trichothecenes, zearalenone, pet food contamination

## Abstract

Veterinary diets are intended for diseased animals and may contain cereal grains, mainly maize and/or wheat. These, in turn, are often infected with pathogens of the *Fusarium* genus, which are able to produce numerous harmful mycotoxins. Forty-two samples of veterinary diets for dogs and cats were analyzed for the presence of *Fusarium* species and mycotoxins. Species were identified using molecular methods and the ergosterol and mycotoxins (fumonisin B_1_, deoxynivalenol, nivalenol and zearalenone) were quantified using HPLC methods. Two *Fusarium* species were identified: *Fusarium proliferatum* and *Fusarium verticillioides*. The highest concentrations of fumonisin B_1_, deoxynivalenol, nivalenol and zearalenone were 74.83, 2318.05, 190.90, and 45.84 ng/g, respectively. Only 9.5% of the samples were free from *Fusarium* mycotoxins. The acceptable limits of mycotoxin content in animal feed, specified by the EU regulations, were not exceeded in any of the samples tested. The mean mycotoxin content in veterinary diets for cats was lower than for dogs. Thus, it is recommended that veterinary diets are examined, since the mycotoxin contamination pose additional risk to animal health. The knowledge on *Fusarium* occurrence in veterinary diets is scarce and as far as we are aware this is the first report concerning the occurrence of *Fusarium* spp. and their important secondary metabolites—mycotoxins—in different types of veterinary diets for companion animals in Poland.

## 1. Introduction

Veterinary diets are being designed and used in the course of treatment or help in treating animals with particular disease. According to the descriptions given on labels, veterinary diets often contain cereals, usually maize, wheat and/or rice, similarly to the regular dog and cat food. Cats and dogs are carnivores and their digestive tract is not well-adapted to digest dietary fiber. Therefore, the digestibility of food is determined by the content of meat—the more meat and less grain it contains, the better is the digestibility. Cereal grain used for the production of pet food can contain mycotoxins produced by microscopic fungi infecting the cereal heads during plant ripening on the field, or after harvest, in case of its improper storage [1,2].

Rice, wheat, and maize are the most important cereals worldwide, which grown in temperate, humid climate (wheat), and temperate, tropical and sub-tropical climate (maize). These climatic conditions are optimal for development of the most damaging cereal pathogens—*Fusarium* fungi. Most species grow in temperatures around 25–35 °C and high humidity [3,4,5]. These fungi cause huge losses in cereal crops every year. The most important *Fusarium* species for economic reasons are *F. verticillioides* and *F. proliferatum* as pathogens of maize, and *F. culmorum*, *F. avenaceum*, and *F. graminearum*—the pathogens of wheat [6]. A parameter used to estimate the contamination of food by the fungal biomass is the ergosterol—a component of fungal cell wall [7]. *Fusarium* biosynthesize significant amounts of secondary metabolites called mycotoxins, which are secreted and accumulated at the infected tissue [8]. Most harmful mycotoxins (e.g., aflatoxins, ochratoxin A, zearalenone) are resistant to high pressures and temperatures and, therefore, are not degraded during technological processes, which means that they are often transferred into the foodstuffs and feedstuffs [9,10,11]. Mycotoxins may be harmful to bacteria, fungi, plants, animals, and humans. The three most important mycotoxin groups produced by *Fusarium* fungi are: fumonisins (FBs), trichothecenes (including deoxynivalenol—DON) and zearalenone—ZON [6]. Fumonisin B_1_ (FB_1_)—produced by *F. verticillioides* and *F. proliferatum*—interferes with the metabolism of sphingolipids and was proven to cause leukoencephalomalacia in horses, pulmonary edema in pigs, and neural tube defects in fetuses [12,13,14]. Trichothecenes disrupt the process of translation, stop the process of DNA and RNA synthesis and cause oxidative stress, disturbing basic processes in the cell. The most prevalent trichothecene is DON (also called vomitoxin), harming the digestive system by causing diarrhea, vomiting, weight loss, and anorexia [15,16]. Zearalenone binds to the estrogen receptors causing changes in genes expression, resulting in disturbing of functioning of the reproductive system and fertility [1,17].

International research communities are well-aware of the need of monitoring the content of mycotoxins in cereal grain, its products—food and feed—with an assessment of their toxicity [18]. Concerning the toxicity to animal, the European Union introduced legally acceptable levels of mycotoxins in feed [19,20]. Although there are studies confirming the negative impact of mycotoxins on dogs’ and cats’ health, similar legal limits for mycotoxin content in the pet foods were not established yet [21,22]. Only in the case of two groups of toxins, there are guidance values relative to a feeding stuff for pet animals (sum of fumonisins B_1_ and B_2_—5000 ng/g) and for cats (sum of T2 and HT2 toxin—50 ng/g) [19,20].

Numerous studies have been conducted on the content of the fungal microflora in animal feeds, dedicated particularly for farm animals (poultry, cattle, pigs) [23,24,25,26,27,28,29]. The most commonly identified fungi in feed and feed components were belonging to *Fusarium*, *Aspergillus*, *Mucor*, *Penicillium*, and *Rhizopus* genera, which explains the presence of various groups of mycotoxins [23,24,25,26,27,28,29]. The presence of fungal microflora and mycotoxins in feeds for livestock has contributed to the development of research focused on the quality and purity of foods for companion animals [19,30,31,32,33,34]. Until now, few studies on feed and food microbiology and the mycotoxin contamination were focused on veterinary diets. Examination of diets appears to be particularly important because weakened organisms of animals struggling with the disease may be more susceptible to the negative effects exerted by mycotoxins then the healthy ones. Few deaths of dogs were documented as directly caused by mycotoxins ingested with pet food. Acute poisonings and deaths of dogs fed with food containing maize infested with toxin-producing fungi were reported in 1951, 1998, and 2005 in the United States [35,36,37]. Similar phenomena occurred in other parts of the world, including India in 1974 and South Africa in 1987 [38,39]. There are known cases when causes of death of dogs were precisely determined. These cases were mostly related to the toxic effects of aflatoxins and ochratoxin A, leading to liver and kidney damage [40,41,42,43].

The risk of mycotoxin contamination in dog and cat food and veterinary diets, as well as the impact of mycotoxin on the health of companion animals is still underexplored. The aim of this study was to evaluate the microbiological quality of veterinary diets for dogs and cats, designed specifically for different types of diseases, as well as to analyze the mycotoxins produced by *Fusarium* species present in the diets.

## 2. Materials and Methods

The material consisted of veterinary diets for dogs and cats, supplied from 16 different producers and available on the Polish market. The samples were obtained in hermetically sealed packages from pet shops, veterinary clinics, as well as from pet owners. In total, the collected research material was represented by 42 samples, which consisted of 17 veterinary diets for cats and 25 veterinary diets for dogs. The weights of the samples ranged from 60 to 150 g. Individual samples were divided into six groups following the criterion of the cereal component: samples containing exclusively (i) maize, (ii) wheat, (iii) rice, (iv) maize and wheat, (v) maize and rice, and (vi) none of these cereals. Veterinary diets were grouped according to the kind of disease: digestive system, urinary system, musculoskeletal system, and allergies and obesity or overweight (Table 1). The veterinary diets for dogs and cats are formulated as croquettes of different sizes, therefore material was homogenized before the analysis. Large croquettes destined for large breed dogs was crushed in a ceramic mortar, and then milled. Milled food was stored at −20 °C until analysis.

### 2.1. Microbiological Fungal Species Identification

About 1g of milled food was scattered evenly over the surface of the potato dextrose agar (PDA) medium plate (two plates per sample, 9 cm of diameter). The plates were incubated for seven days at room temperature. The growing mycelia were passaged onto fresh PDA plates until obtaining pure isolates. Then mycelia of all isolated fungi were transferred to synthetic nutrient agar (SNA) medium plates. The plates were incubated at room temperature for four to six weeks. Macroconidia were viewed microscopically using light microscope (Olympus BX40, Hamburg, Germany) at 200× magnification (10× objective lens magnification, 20× ocular lens magnification) and fungi were identified to a genus level.

### 2.2. Molecular Analysis

For genomic DNA extraction, the mycelia were frozen in liquid nitrogen and then homogenized in a plastic mortar. Cell lysis was performed using a buffer consisting of 800 µL cetyltrimethylammonium bromide solution and 4 µL β-mercaptoethanol, and 150 µL mixture of chloroform:octanol (24:1, *v*/*v*). Samples were incubated in a water bath at 65 °C for 20 min, then cooled down to room temperature. Subsequently, 150 µL of chloroform:octanol (24:1, *v*/*v*) mixture was added and centrifuged (12,000 rpm, 15 min, 4 °C). The supernatants (600 µL) were transferred to 1.5 mL tubes, 60 µL of 3 M sodium acetate (pH = 5.4) and 800 µL of cold 96% ethanol were added. Samples were incubated for 20 min at −20 °C. The samples were air-dried, and then DNA pellets were re-dissolved in 150 µL of TE (Tris-EthylDimethylTetraAcecic) acid buffer (pH = 8.0).

Polymerase chain reaction was performed using primers Tef1R and Ef728M used previously [44]. Reaction mixture composition was as follows: sterile water—20.5 µL, reaction buffer (Finnzymes, Espoo, Finland)—2.5 µL, dNTP mix—1.5 µL, primers—0.2 µL, Taq DNA polymerase (Finnzymes, Espoo, Finland)—0.2 µL. PCR was started with an initial denaturation (94 °C for 5 minutes), followed by 35 cycles (94 °C for 50 s, 58 °C for 50 s, 72 °C for 1 min) and final elongation (5 min at 72 °C). Amplified PCR products were electrophoresed using 1.5% agarose gels in TBE buffer, the fragment sizes were estimated using 1 kb standard DNA Ladder Plus (Fermentas, Waltham, MA, USA). Electrophoresis was performed in BioRad Sub-Cell DNA96 chambers (Hercules, CA, USA) for 90 minutes at a voltage of 90 V and 150 mA intensity, fragments were stained using GelRed dye (Biotium, Fremont, CA, USA) and visualized by UV transilluminator (λ = 302 nm; Syngen, Wrocław, Poland).

The enzymatic purification was carried out in 0.85 µL reaction mixture consisting of 0.15 µL sterile distilled water, 0.3 µL exonuclease I (Epicentre, Madison, WI, USA) and 0.45 µL alkaline phosphatase SAP (Promega, Madison, WI, USA). Eight µL of mineral oil and 3.3 µL of PCR product were added to the mixture. The following program was used: 10 min at 37 °C and 15 min at 80 °C.

Fluorescent labeling was performed using 0.5 μL of Ef728M primer and 1.3 μL of ABI Prism BigByeTerminator v3.1 Cycle Sequencing Kit (Applied Biosystems, Foster City, CA, USA) according to the manufacturer’s instruction.

After fluorescent labeling the PCR products were precipitated with 13.5 μL of 96% ethanol with sodium acetate for 15 min on ice and then centrifuged for 20 minutes at 10,500 rpm. The supernatant was removed and 200 μL of 70% ethanol were added. The samples were centrifuged at 10,500 rpm for 6 min and again for 1 min after removing 180 μL of the supernatant. Then the samples were air-dried. Sequence reading was done by Laboratory of DNA Sequencing and Oligonucleotide Synthesis IBB PAS in Warsaw. Molecular identification of fungal species was done by comparing the sequences to the reference sequences deposited in the NCBI GenBank database using the BLASTn algorithm.

### 2.3. Ergosterol and Mycotoxins Analysis

#### 2.3.1. Standards and Chemical Reagents

Ergosterol (ERG), fumonisin B_1_ (FB_1_), zearalenone (ZON), deoxynivalenol (DON), and nivalenol (NIV) standards were purchased with a standard grade certificate (purity above 98%) from Sigma-Aldrich (Steinheim, Germany). Stock solutions of standards were prepared in acetonitrile except ERG in methanol at 1.0 mg/mL concentrations and stored at −20 °C. Sodium dihydrophosphate, potassium hydroxide, sodium hydroxide, potassium chloride, acetic acid, hydrochloric acid, and *o*-phosphoric acid were purchased from POCh (Gliwice, Poland). Organic solvents (HPLC grade), disodium tetraborate, n-pentane, 2-mercaptoethanol, sodium acetate, and all the other chemicals were also purchased from Sigma-Aldrich (Steinheim, Germany). Water for the HPLC mobile phase was purified using a Milli-Q system (Millipore, Bedford, MA, USA).

#### 2.3.2. Extraction and Purification Procedure

##### Ergosterol

Samples (each in triplicate) containing 100 mg of ground material were suspended in 2 mL methanol in a culture tube, treated with 0.5 mL of 2 M aqueous sodium hydroxide and sealed tightly. Samples were irradiated twice in a microwave oven (370 W) for 20 s according to Waśkiewicz et al. 2014 [45]. After 15 min contents of cultures tubes were neutralized with 1 M aqueous hydrochloric acid, then 2 mL of methanol were added and samples were extracted with pentane (3 × 4 mL). The combined pentane extracts were evaporated to dryness in a stream of nitrogen, before analysis dissolved in 1 mL of methanol and 10 µL of thus prepared mixture were analyzed by HPLC.

##### Deoxynivalenol, Nivalenol, and Zearalenone

Ground samples (10 g) were soaked with 30 mL of acetonitrile:water (80:20, *v*/*v*) and mycotoxins were extracted overnight. After filtration through a Filtrak 389 paper (Munktell & Filtrak GMBH, Barenstein, Germany), 10 mL of the extract were collected, diluted with 40 mL of water and filtered through a Whatman No. 5 paper (Whatman International Ltd, Maidstone, UK). Half of the extract was used for DON and NIV analysis, while the remaining part was used for ZON determination. The first part of the extract was applied on top of an immunoaffinity DON-NIV column, while the second part was applied on the top of the Zearala Test column (IAC, Vicam, Milford, MA, USA) and then proceeding in accordance with the manufacturer’s procedure.

##### Fumonisin B_1_

10 g of homogenized ground samples of veterinary diet were homogenized for 3 min in 30 mL of methanol-water (3:1, *v*/*v*) and filtered through Whatman no. 4 filter paper (Whatman International Ltd, Maidstone, UK) according to Stępień et al. 2011 [46]. The extract was adjusted to a pH = 5.8–6.3 using 0.1 M KOH. A SAX cartridge was attached to the SPE manifold unit (Supelco, Bellefonte, PA, USA) and conditioned at a flow rate of 2 mL/min successively with 5 mL of methanol, followed by 5 mL of methanol:water (3:1, *v*/*v*). An aliquot (5 mL) of the filtered subsample extract was applied at the top of the conditioned cartridge at a flow rate of 2 mL/min, washed with 8 mL methanol:water (3:1, *v*/*v*), immediately followed by 3 mL of methanol. Fumonisin B_1_ was eluted from the column with 10 mL of 1% acetic acid in methanol. The elute was evaporated to dryness at 40 °C under a stream of nitrogen. Dry residue was stored at −20 °C until HPLC analyses.

#### 2.3.3. HPLC Analysis

The chromatographic system consisted of Waters 2695 high-performance liquid chromatograph (Waters, Milford, USA) with detectors:

- Waters 2996 Photodiode Array Detector with Nova Pak C-18 column (150 × 3.9 mm) and methanol:acetonitrile (90:10, *v*/*v*) as a mobile phase for ERG (λ_max_ = 282 nm) analysis and with Nova Pak C-18 column (300 × 3.9 mm) and methanol:water (25:75, *v*/*v*) as a mobile phase for DON and NIV analysis (λ_max_ = 224 nm),
- Waters 2475 Multi λ Fluorescence Detector (λ_ex_ = 274 nm, λ_em_ = 440 nm) and Waters 2996 Photodiode Array Detector with Nova Pak C-18 column (150 × 3.9 mm) and acetonitrile:water:methanol (46:46:8, *v*/*v*/*v*) as a mobile phase for ZON analysis,
- Waters 2475 Multi λ Fluorescence Detector (λ_ex_ = 335 nm, λ_em_ = 440 nm) with an XBridge column (3.0 × 100 mm) and methanol:sodium dihydrogen phosphate (0.1 M in water) solution (77:23, *v*/*v*) adjusted to pH 3.35 with o-phosphoric acid as a mobile phase for FB1 analysis after pre-column derivatization with o-phthaldialdehyde (OPA) reagent.

The linearity of the standard curves was 0.9897, 0.9991, 0.9990, 0.9989, and 0.9982 for FB_1_, ZON, DON, NIV, and ERG, respectively. The limits of quantification (LOQ) were (in ng/g): 1.0 for ZON, 2.0 for FB_1_ and 10.0 for DON, NIV, and ERG. The recovery experiment was performed on mycotoxin—free veterinary diet samples, spiked with three different levels of each mycotoxin separately at a concentration of 5.0, 8.0, and 10.0 ng/g for FB_1_ and ZON and 20.0, 40.0, and 80 ng/g for DON and NIV. On the basis of these experiments, recovery rates and standard deviations (RSD) were calculated. For the majority of the tested mycotoxins, the recovery rates ranged between 88 and 109% (depending on the matrix), whereas the RSD values did not exceed 13%.

### 2.4. Statistical Analysis

Firstly, the normality of distributions for studied traits was tested using the Shapiro–Wilk normality test [47]. One-way analysis of variance (ANOVA) was carried out to determine the effects of type of diet on the variability of ergosterol (ERG), fumonisin B_1_ (FB_1_), zearalenone (ZON), deoxynivalenol (DON), and nivalenol (NIV). Mean values and standard deviations of individual characteristics were calculated. Least significant differences (LSDs) were calculated for individual characteristics and on this basis homogeneous groups were determined. Significance of differences was estimated at α = 0.05 using the Student’s *t*-test. The relationships between the ERG, FB_1_, ZON, DON, and NIV were determined using the simple correlation analysis. Relationships of five observed traits were presented in the form of the scatterplot matrix. Data analysis was performed with the GenStat 17 package.

## 3. Results

### 3.1. Microbiological and Molecular Fungal Species Identification

**Figure 1:**
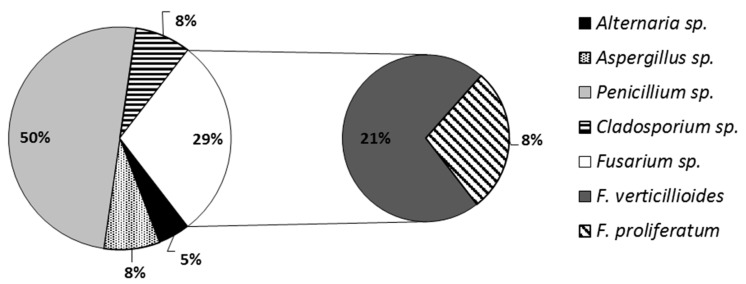
Percentage of fungi isolates identified in veterinary diets.

Veterinary diet samples were divided into five groups, depending on the type of disease (allergies, obesity, excretory, digestive, and skeletal system diseases). All of them were screened for the presence of filamentous fungi (Figure 1). Taking together, *Penicillium* sp. was the prevailing genus (43 isolates) among 86 isolates, followed by *Fusarium verticillioides* (18 isolates). Four isolates belonged to *Alternaria* sp., seven isolates were identified as *Cladosporium* sp. and another seven as *Aspergillus* sp. There were nine isolates of *Fusarium proliferatum* (Figure 1). Virtually all categories of diets were contaminated with mycotoxin producers—*F. proliferatum* and *F. verticillioides*, however, there were three categories contaminated with higher frequencies than the others: for obesity management, excretory and digestive system diseases (Table 1). The full information on the fungal genera observed in the diets of various composition was given in the Supplementary Table S1.

### 3.2. Ergosterol and Mycotoxins Quantification

In the experimental material ergosterol (ERG) and *Fusarium* mycotoxins (zearalenone, deoxynivalenol, nivalenol, and fumonisin B_1_) were analysed. Ergosterol as a fungal bioindicator was found in all tested samples, at various mean concentrations ranging from 0.05 (allergies) to 6.82 µg/g (excretory system diseases) (Table 2). 

**Figure 2:**
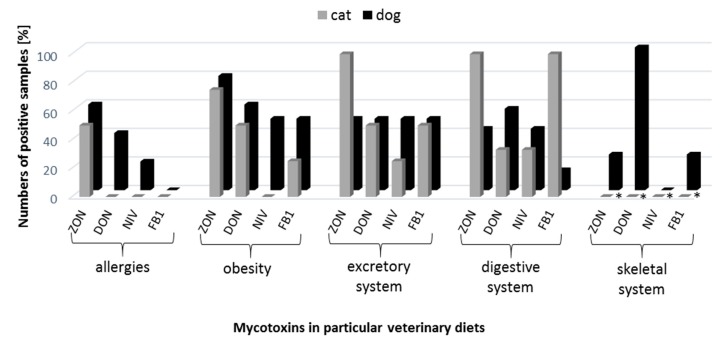
Number of positive samples [%] containing mycotoxins present in veterinary diets for dogs and cats. * there were no diets for cats present in this group.

Among all mycotoxins, ZON was detected in veterinary diet samples with the highest frequency (69%), followed by DON (52%), FB_1_ (33%) and NIV (26%) (Figure 2). Taking into account the division into dog and cat veterinary diets, ZON and FB_1_ were more frequently observed in cat food (88% and 47%, respectively), whereas DON and NIV in dog food (60% and 32%, respectively) (Figure 2). Even though the incidence of zearalenone was the highest, the concentrations were the lowest, at 1.22–51.70 ng/g (Table 2). In the case of other mycotoxins (DON, NIV, FB_1_) at the lowest frequency we observed higher levels of these compounds: 24.87–2415.03, 17.43–200.40, and 4.89–80.13 ng/g for DON, NIV, and FB_1_, respectively (Table 2). With regard to the type of disease, it was shown that diets for allergies were ‘the safest’ and free from mycotoxins, except for ZON (Table 1). In addition, the highest level of ergosterol (mean 2.57 µg/g) and relatively high concentrations of mycotoxins (especially for FB_1_—49.50 ng/g) were found in diet samples for excretory system diseases (subgroup kidney diseases), which was also related to their high frequency. The highest concentrations of mycotoxins were found in veterinary diets dedicated for allergies (51.70 ng/g—ZON), excretory system diseases (80.13 ng/g—FB_1_) and overweight (2415.03 ng/g—DON and 200.40 ng/g—NIV) (Table 2).

Comparing our results to the acceptable limit of DON mycotoxin in pig feeds (900 ng/g, no limit for cat’s and dog’s food), deoxynivalenol concentration was exceeded only in one sample—dog veterinary diet for overweight (2415.03 ng/g) (Table 2).

When analyzing the results, we took into account the share of cereal components in the veterinary diets: one component—wheat or rice or maize, two components—maize and rice or maize and wheat, and three components—wheat, rice, and maize (Table 3 and Table 4). Moreover, some samples contained no cereal component (4.8%). Most of the samples contained two cereal components—40.5 and 16.7% for maize and rice, and maize and wheat, respectively. High mycotoxin concentrations were recorded in these groups. In addition, there were two records with the highest levels of ZON and FB_1_ among veterinary diet samples for digestive system diseases—8.96 and 41.64 ng/g, respectively, and of FB_1_ in the diet for excretory system diseases—74.83 ng/g (Table 2, Supplementary Table S2). It was also common that samples containing all cereals (wheat, rice, and maize) were associated with the simultaneous presence of *Fusarium* fungi.

Statistical analysis demonstrated a positive correlation between ergosterol level and nivalenol concentration in veterinary diet samples (Table 5). For other mycotoxins there was no correlation with ERG contents.

## 4. Discussion

Cereals are usually the dominant components of dry foods and veterinary diets of companion animals, being used as ‘fillers’, supposed to improve the texture of croquettes. Thus, they are the main source of contamination with fungal microflora [48]. Fungi can not only reduce the nutritional value of the feed but also be harmful to the health of animals through the biosynthesis of various mycotoxins [49,50,51,52]. Research studies on the microbiological quality of pet foods were carried out by several scientists [31,33,53,54,55]. *Aspergillus* was the dominant genus in all of these experiments. In studies conducted by Martins et al. (2003) and Błajet-Kosicka et al. (2014) *Aspergilli* were followed by *Penicillium* and *Mucor* species [31,33]. Campos et al. (2008) and Copetti et al. (2009) obtained similar results to those of the present study, where *Aspergillus*, *Penicillium*, and *Fusarium* were the prevailing species of fungi [54,55]. All these fungal genera (except for *Mucor*) are toxigenic [56]. Mycological contamination was investigated in feeds for other animals, mainly for poultry, confirming that *Penicillium*, *Mucor*, *Rhizopus*, *Aspergillus*, and *Fusarium* spp. dominate also in animal feed, regardless of the region where the studies were conducted [24,29,48,52,57].

*Fusarium* species identified during our research are often responsible for causing fusariosis in cereals and ear rot of maize. These include two cereal species which are most frequently used in the production of pet food, i.e., wheat (infected mostly by *F. culmorum*) and maize (associated with *F. verticillioides* and *F. proliferatum*) [58,59,60,61]. It is highly possible that cereal grain infected with *Fusarium* fungi was the primary source of veterinary diet contamination with mycotoxins.

Currently, there are no regulations that specifically limit the values of fungal microflora in food and feed, but Greco et al. (2014) applied a method of discrimination proposed by Gimeno (2002), which classified a sample containing below 3 × 10^4^ CFU/g as being of high quality, while a sample containing over 7 × 10^4^ CFU/g as low quality [57,62]. According to this criterion, the microbial quality of samples examined by Copetti et al. (2009), Kubizna et al. (2011) and Martins et al. (2003) can be defined as good, because the values did not exceed 10^2^ CFU/g [33,55,63].

The levels of fungal contamination are often measured by the amount of ergosterol in the various cereal samples and their products. This parameter, however, has its drawbacks because of the food production process, when the substrate is subjected to the extrusion (high pressure and temperature) which in fact means sterilization. Unfortunately, none of the researchers attempted to examine the ergosterol content in animal feeds, but there are many papers available describing this parameter in cereals and their products, proving the correlation between the content of ergosterol and microbiological quality [64,65,66]. It is assumed that the concentration of ergosterol lower than 3 μg/g confirms a good product quality and, in our study, only 17% of samples exceeded this level [67]. In agricultural research, it is assumed that acceptable ERG level cannot exceed 7.0 µg/g [68]. Our studies indicated that ergosterol concentrations were below this limit. The low amounts of ergosterol detected in the veterinary diets suggest high quality of cereal grain used for their production. Ergosterol content is an indicator of the total content of the fungal biomass including toxigenic and non-toxigenic fungi, therefore, high concentration of ergosterol does not necessarily correlate to the content of mycotoxins [66].

Most of the studies on the content of mycotoxins in feeds were related to dog food and reported their contamination with fumonisins (mainly fumonisin B_1_), which were followed by deoxynivalenol and zearalenone [32,33,34,69,70,71,72,73,74]. Only Böhm et al. [70] and Gazzotti et al. [32] examined a wide range of *Fusarium* mycotoxins (DON, ZON, fumonisins) in dry dog food. This relates probably to different methodology used during quantification of these compounds and possible difficulties in discussing the additive effects of simultaneous action of mycotoxins. Besides dog food, cat food samples have been investigated by Martins et al. [33] and Scudamore et al. [34], who studied FB_1_ content. Results obtained by Scudamore et al. [34] showed that FB_1_ contamination was lower (105 ng/g) in dog food than in cat food (690 ng/g), which does not correspond to our results, where the average content of FB_1_ in dog food (33.51 ng/g) was higher than in cat food (29.54 ng/g), however, the difference was not statistically significant. Similar average FB_1_ content was observed by Martins et al. [33]—17.3 ng/g, while the highest average value was described by Mulunda et al. [72]—1556 ng/g, which means that at the acceptable daily intake of fumonisins (20 μg/kg b.w./day) present in these foods can pose a serious threat to pet health [72].

The lowest average content of ZON has been described by Hołda and Głogowski [71]: 3.11 ng/g, and the highest—by Böhm et al. (2010)—80 ng/g [70]. The highest concentration of ZON in the sample was found by Bissoqui et al. [68] and it was equal to 442.2 ng/g. Gazzotti et al. [32] observed similar contamination of premium dog food (10 ng/g) to those presented in the our study (8.14 ng/g). According to Bissoqui and co-workers [68] acceptable dietary intake of ZON for pets was 1.00 μg/kg b.w./day [32,69,70,71].

DON content in dog food was investigated and found in the amounts of 2.7, 116.0, 103.0, 308.0, and 483.0 ng/g, respectively [32,70,71,72,73]. The results of Böhm et al. (2010) corresponded to our examination of the veterinary diets for cats (113.78 ng/g) [70]. Daily dose of DON higher than 4500 and 7700 ng/g food may cause disorders of food intake in dogs and cats, respectively [75]. So far—despite the proved toxicity—there have been no reports regarding the contamination of pet food with nivalenol [76].

Among available literature only Zwierzchowski and co-workers [74] raised the issue of mycotoxin contamination of veterinary diets, called ‘therapeutic diets’, in which the content of zearalenone and its derivatives was studied. The mean content of ZON in the diets was 34.04 ng/g and was slightly lower than in food (41.14 ng/g), but was higher than in our results (10.89 ng/g). The highest contamination of veterinary diets was recorded for the one intended for kidney disease—158 ng/g, and exceed about three times the concentration for ZON obtained in our research [74].

Maize and its pathogens prevailed in veterinary diets studied but their presence in the sample was not always associated with the occurrence of the respective mycotoxins. It might be due to environmental conditions which determine the production of mycotoxins by *Fusarium*, and which could be far from optimal ones (15–30 °C and 0.98 aw) [5,60]. It may also be caused by the fact that contamination occurred after harvesting, so the *Fusarium* fungi did not have enough time or appropriate conditions to produce detectable amounts of toxins [5,50]. Trichothecenes and zearalenone were detected in veterinary diets samples but there were no fungi responsible for their production, i.e., *F. culmorum* and/or *F. graminearum*. It could be explained by the extrusion process, which results in fungal cells’ death but does not cause degradation of mycotoxins, which are resistant to high temperature and pressure [5,9].

## 5. Conclusions

The contents of mycotoxins measured during the present study did not exceed the permissible levels recommended by the European Commission for animal feed [19,20]. It should be taken into account that dry food is currently used as main component of pet feeding for most of their lives. It means that a long-term intake of medium or high doses of mycotoxins in pet foods may entail adverse health effects, because these compounds are accumulated in animals’ tissues [77]. It is not clear yet how the health is affected by mycotoxins’ co-occurrence. De Souza and Scussel [78] showed that the administration of dog food naturally contaminated with mycotoxins deteriorated the animal health status, causing dysfunction of many organs, particularly liver and kidneys [78]. Veterinary diets are applied for shorter periods of time but the treated animals are weakened and can be more sensitive to mycotoxins’ influence. The susceptibility of diseased animals to mycotoxins present in veterinary diets should be examined in detail during future studies. The reduction of cereals content (mainly wheat and maize) in pet foods and veterinary diets lowers the probability of contamination with mycotoxins. In addition, the rice in the diet of animals is less harmful than maize and wheat, thus being a good alternative for replacing the most popular cereals in pet food products [79]. On the basis of available literature, this is the first report concerning the presence of *Fusarium* spp. and mycotoxins in veterinary diets for dogs and cats.

## Figures and Tables

**Table 1:** Characteristics of the cereal components present in veterinary diets for dogs and cats.

Specification of Diet	Sample No.	Cereal Component		
		Rice	Maize	Wheat
Allergies	cD21	+	-	-
	cD22	+	-	-
	dD34	+	-	-
	dD35	+	-	-
	dD68	-	-	-
	dD69	-	-	-
	dD77	+	-	-
Overweight/obesity	cD3	-	-	+
	cD10	+	+	-
	cD11	+	+	-
	cD20	+	+	-
	dD31	-	+	+
	dD32	-	+	+
	dD62	+	+	-
	dD64	-	+	+
	dD74	-	+	-
Excretory system diseases	cD5	-	+	-
	cD7	+	+	+
	cD8	+	+	-
	cD9	-	+	+
	cD17	+	+	-
	cD19	+	+	-
	cD27	+	+	+
	cD28	+	-	-
	dD38	-	+	+
	dD44	-	+	+
	dD61	+	+	-
	dD73	+	+	-
Digestive system diseases	cD6	+	+	+
	cD18	+	+	-
	cD23	+	+	-
	dD37	-	+	+
	dD63	+	+	-
	dD66	-	+	-
	dD70	-	-	+
	dD71	+	+	-
	dD72	+	-	-
	dD75	+	+	-
Skeletal system diseases	dD41	+	+	+
	dD65	+	+	-
	dD67	+	+	-
	dD76	+	+	-

c—cat, d—dog, D—diet.

**Table 2:** Contents of *Fusarium* fungi, ergosterol (μg/g) and mycotoxins (ng/g) in samples of veterinary diets for dogs and cats.

Specification of Diet	Sample No.	*Fusarium*		ERG [μg/g]	Mycotoxins (ng/g)			
		*Fp*	*Fv*		ZON	DON	NIV	FB_1_
Allergies								
Food allergy	cD21	-	-	1.04 ± 0.06	n.d.	n.d.	n.d.	n.d.
	cD22	-	-	0.79 ± 0.08	1.71 ± 0.43	n.d.	n.d.	n.d.
	dD34	-	+	0.42 ± 0.03	45.84 ± 5.79	n.d.	n.d.	n.d.
	dD35	-	-	0.06 ± 0.01	11.88 ± 3.24	n.d.	n.d.	n.d.
	dD69	-	-	0.32 ± 0.03	n.d.	61.90 ± 3.48	38.92 ± 8.68	n.d.
Skin allergy	dD77	-	-	2.44 ± 0.21	n.d.	n.d.	n.d.	n.d.
Other allergy	dD68	-	+	0.12 ± 0.01	2.07 ± 0.40	185.40 ± 19.01	n.d.	n.d.
Overweight/obesity								
Obesity management	cD3	-	-	0.36 ± 0.01	n.d.	n.d.	n.d.	n.d.
	cD10	-	+	3.03 ± 0.13	24.81 ± 4.64	n.d.	n.d.	n.d.
	cD11	-	-	1.46 ± 0.03	7.79 ± 0.28	n.d.	n.d.	n.d.
	cD20	-	+	2.16 ± 0.10	7.64 ± 0.34	351.02 ± 17.82	n.d.	5.02 ± 0.12
	dD31	-	-	1.10 ± 0.17	7.72 ± 1.28	n.d.	n.d.	n.d.
	dD32	-	+	0.57 ± 0.04	7.80 ± 0.36	n.d.	21.33 ± 4.11	15.84 ± 4.43
	dD62	-	+	0.72 ± 0.03	6.41 ± 1.03	103.33 ± 11.38	190.90 ± 12.42	59.05 ± 2.93
	dD64	+	-	0.67 ± 0.05	1.78 ± 0.34	257.68 ± 14.04	n.d.	n.d.
	dD74	-	-	1.63 ± 0.08	n.d.	2318.05 ± 93.43	n.d.	n.d.
Excretory system diseases								
Renal system diseases	cD7	-	-	3.05 ± 0.11	2.71 ± 0.39	n.d.	n.d.	n.d.
	cD9	+	+	1.94 ± 0.07	7.86 ± 0.39	n.d.	n.d.	n.d.
	cD17	-	-	4.79 ± 0.20	3.92 ± 1.09	n.d.	n.d.	n.d.
	cD28	-	-	6.57 ± 0.23	9.05 ± 1.76	n.d.	n.d.	n.d.
	dD38	+	+	1.99 ± 0.17	n.d.	n.d.	n.d.	42.40 ± 4.08
Kidney diseases	cD5	-	+	2.26 ± 0.12	4.32 ± 0.37	53.53 ± 3.82	n.d.	21.50 ± 3.28
	cD8	+	+	2.03 ± 0.07	19.99 ± 1.42	116.67 ± 5.49	n.d.	26.29 ± 5.44
	cD19	-	+	2.95 ± 0.09	6.34 ± 0.47	79.69 ± 7.12	n.d.	70.02 ± 3.92
	cD27	+	+	0.80 ± 0.01	2.25 ± 0.26	188.62 ± 13.41	37.25 ± 4.55	74.83 ± 5.02
	dD44	+	-	3.15 ± 0.17	n.d.	n.d.	39.82 ± 1.35	61.94 ± 5.54
	dD61	-	+	0.29 ± 0.02	1.95 ± 0.20	448.35 ± 16.78	86.23 ± 4.30	n.d.
	dD73	-	-	0.99 ± 0.05	1.51 ± 0.26	93.93 ± 6.66	n.d.	n.d.
Digestive system diseases								
Intestinal tract diseases	cD18	-	+	4.02 ± 0.09	1.77 ± 0.24	n.d.	n.d.	41.22 ± 4.21
	dD63	+	-	0.43 ± 0.03	n.d.	191.75 ± 9.93	n.d.	14.50 ± 2.89
	dD66	-	-	0.58 ± 0.03	1.90 ± 0.41	27.76 ± 2.68	106.29 ± 10.43	n.d.
	dD72	-	+	1.44 ± 0.14	3.16 ± 0.27	296.13 ± 7.37	53.98 ± 2.89	n.d.
	dD75	-	-	0.99 ± 0.04	n.d.	94.85 ± 10.19	n.d.	n.d.
Liver diseases	cD6	-	+	2.62 ± 0.16	8.96 ± 1.13	41.53 ± 4.66	n.d.	41.64 ± 5.39
	cD23	-	+	4.59 ± 0.22	7.14 ± 0.34	n.d.	28.28 ± 3.32	23.46 ± 3.82
	dD37	-	-	0.31 ± 0.02	n.d.	n.d.	n.d.	n.d.
	dD70	-	-	0.29 ± 0.03	n.d.	n.d.	n.d.	n.d.
	dD71	-	-	4.88 ± 0.15	3.37 ± 0.58	n.d.	20.06 ± 2.58	n.d.
Skeletal system diseases								
Bone disorders	dD41	-	+	0.60 ± 0.05	n.d.	140.63 ± 16,63	n.d.	n.d.
	dD67	-	-	1.07 ± 0.07	n.d.	37.23 ± 4.55	n.d.	16.66 ± 1.42
	dD76	-	-	0.53 ± 0.03	40.07 ± 3.06	394.86 ± 8.55	n.d.	n.d.
Teeth	dD65	-	-	0.60 ± 0.03	n.d.	42.23 ± 4.62	n.d.	n.d.

c—cat, d—dog, D—diet, n.d.—not detected, *Fv—Fusarium verticillioides*, *Fp—Fusarium proliferatum*.

**Table 3:** Minimum, maximum, and average values of ergosterol in veterinary diets. Diets were grouped based on the diseases, consecutive upper superscript letters mark groups which differ significantly.

Specification of Diet	ERG [μg/g]		
	Minimum	Maximum	Mean ± s.d.
Allergies	0.05	2.68	0.74 ± 0.79 ^c^
Obesity/overweight	0.35	3.16	1.30 ± 0.84 ^bc^
Excretory system diseases	0.27	6.82	2.57 ± 1.70 ^a^
Digestive system diseases	0.25	5.04	2.01 ± 1.79 ^ab^
Skeletal system diseases	0.51	1.13	0.70 ± 0.23 ^c^
LSD_0.05_	0.90		

**Table 4:** Minimum, maximum, and average values of ZON and FB_1_, and DON and NIV in veterinary diets. Diets were grouped based on the diseases, consecutive upper superscript letters mark groups which differ significantly.

Specification of Diet	ZON [ng/g]			FB1 [ng/g]			DON [ng/g]			NIV [ng/g]		
	Min	Max	Mean ± s.d.	Min	Max	Mean ± s.d.	Min	Max	Mean ± s.d.	Min	Max	Mean ± s.d.
Allergies	1.22	51.7	15.38 ± 19.07 ^b^	n.d.	n.d.	n.d.	59.61	202.58	123.65 ± 68.7 ^b^	30.18	47.6	38.92 ± 8.68 ^b^
Obesity/overweight	1.49	30.16	9.14 ± 7.05 ^c^	4.89	61.29	26.64 ± 24.90 ^b^	26.43	2415.03	611.89 ± 891.47 ^a^	17.71	200.4	106.12 ± 93.24 ^a^
Excretory system diseases	1.25	21.38	5.99 ± 5.40 ^c^	18.84	80.13	49.50 ± 21.73 ^a^	49.81	463.22	163.46 ± 138.25 ^b^	32.23	90.9	53.68 ± 20.94 ^b^
Digestive system diseases	1.54	10.15	4.38 ± 2.83 ^c^	11.27	47.29	30.21 ± 12.70 ^b^	24.87	303.47	130.40 ± 104.6 ^b^	17.43	118.3	52.15 ± 35.51 ^b^
Skeletal system diseases	36.55	42.14	40.07 ± 3.00 ^a^	15.45	18.22	16.66 ± 1.42 ^b^	33.18	404.52	153.73 ± 151.8 ^b^	n.d.	n.d.	n.d.
	LSD_0.05_	5.69		LSD_0.05_	13.3		LSD_0.05_	294.2		LSD_0.05_	31.6	

**Table 5:** Correlations between ergosterol and particular mycotoxins.

	ERG	ZON	DON	NIV	FB_1_
**ERG**	1	−0.115	0.096	−0.408 *	0.099
**ZON**		1	0.281	−0.022	−0.183
**DON**			1	−0.203	−0.271
**NIV**				1	0.343
**FB_1_**					1
* *p* < 0.05					

## Supplementary Materials

The following are available online at http://www.mdpi.com/2076-2607/7/1/26/s1, Table S1: Individual measurements of mycotoxin contents in veterinary diet samples studied.
