# Supplementary material for: Fusarium Species and Mycotoxins Contaminating Veterinary Diets for Dogs and Cats
Source: Microorganisms. 2019 Jan 21;7(1):26. doi: 10.3390/microorganisms7010026 (PMC6352256; doi:10.3390/microorganisms7010026)
Supplement: Supplementary file 1 [file microorganisms-07-00026-s001.pdf]

Supplementary Material

Table 1. Number of all fungal isolates obtained from diet samples.

**Specification of diet**	**Sample No.**	**Fungal genus**				
		***Altermaria* sp.**	***Aspergillus* sp.**	***Cladosporium* sp.**	***Penicillium* sp.**	***Fusarium* sp.**
Allergies	cD21	n.d.	n.d.	n.d.	n.d.	n.d.
	cD22	n.d.	n.d.	n.d.	n.d.	n.d.
	dD34	n.d.	1	1	1	1
	dD35	n.d.	n.d.	n.d.	2	n.d.
	dD68	n.d.	n.d.	n.d.	n.d.	1
	dD69	n.d.	n.d.	n.d.	1	n.d.
	dD77	n.d.	n.d.	n.d.	2	n.d.
Overweight/obesity	cD3	n.d.	n.d.	n.d.	n.d.	n.d.
	cD10	n.d.	n.d.	n.d.	1	1
	cD11	n.d.	n.d.	n.d.	n.d.	n.d.
	cD20	n.d.	1		1	1
	dD31	n.d.	n.d.	n.d.	1	n.d.
	dD32	n.d.	n.d.	n.d.	1	1
	dD62	2	n.d.	1	1	1
	dD64	n.d.	n.d.	n.d.	1	1
	dD74	n.d.	n.d.	n.d.	1	n.d.
Excretory system diseases	cD5	1	n.d.	n.d.	1	1
	cD7	n.d.	n.d.	n.d.	1	n.d.

	cD8	n.d.	n.d.	n.d.	2	2
	cD9	n.d.	n.d.	n.d.	1	2
	cD17	n.d.	1	n.d.	n.d.	n.d.
	cD19	n.d.	1	n.d.	1	1
	cD27	n.d.	n.d.	n.d.	2	2
	cD28	n.d.	n.d.	1	1	n.d.
	dD38	n.d.	n.d.	1	2	2
	dD44	n.d.	n.d.	n.d.	1	1
	dD61	n.d.	n.d.	n.d.	1	1
	dD73	n.d.	n.d.	n.d.	1	n.d.
Digestive system diseases	cD6	n.d.	n.d.	n.d.	n.d.	1
	cD18	n.d.	2	n.d.	2	1
	cD23	n.d.	n.d.	n.d.	1	1
	dD37	n.d.	n.d.	n.d.		n.d.
	dD63	n.d.	1	n.d.	n.d.	1
	dD66	n.d.	n.d.	1	1	n.d.
	dD70	n.d.	n.d.	n.d.	1	n.d.
	dD71	n.d.	n.d.	n.d.	1	n.d.
	dD72	n.d.	n.d.	1	3	1
	dD75	n.d.	n.d.	n.d.	1	n.d.
Skeletal system diseases	dD41	1	n.d.	n.d.	1	1
	dD65	n.d.	n.d.	n.d.	1	n.d.
	dD67	n.d.	n.d.	n.d.	2	n.d.
	dD76	n.d.	n.d.	1	2	n.d.
Summary		4	7	7	43	25

n.d. – not detected

Table S2. Detailed results of ERG and mycotoxin content in veterinary diets.

Specification	Sample No.	Cereal component			ERG [µg/kg]	Mycotoxins [ng/g]				*Fusarium*	
		maize	wheat	rice		ZON	DON	NIV	FB1	*Fv*	*Fp*
Allergies	cD21	-	-	+	1021.20	n.d.	n.d	n.d	n.d.	-	-
					993.25	n.d.	n.d	n.d	n.d.		
					1105.38	n.d.	n.d	n.d	n.d.		
	cD22	-	-	+	859.99	1.22	n.d	n.d	n.d.	-	-
					805.44	2.03	n.d	n.d	n.d.		
					711.27	1.88	n.d	n.d	n.d.		
	dD34	-	-	+	390.54	40,13	n.d	n.d	n.d.	+	-
					442.42	45.69	n.d	n.d	n.d.		
					412.75	51.70	n.d	n.d	n.d.		
	dD35	-	-	+	60.14	9.13	n.d	n.d	n.d.	-	-
					53.33	11.06	n.d	n.d	n.d.		
					71.46	15.45	n.d	n.d	n.d.		
	dD68	-	-	-	107.83	1.71	202.58	n.d	n.d.	+	-
					115.22	2.51	188.65	n.d	n.d.		
					96.66	1.99	164.98	n.d	n.d.		
	dD69	-	-	-	353.33	n.d.	65.90	39.03	n.d.	-	-
					313.29	n.d.	59.61	47.55	n.d.		
					299.82	n.d.	60.18	30.18	n.d.		
	dD77	-	-	+	2683.30	n.d.	n.d.	n.d.	n.d.	-	-
					2280.62	n.d.	n.d.	n.d.	n.d.		
					2352.47	n.d.	n.d.	n.d.	n.d.		
Obesity / overweight	cD3	-	+	-	346.66	n.d.	26.43	n.d.	n.d.	-	-
					371.21	n.d.	32.55	n.d.	n.d.		
					359.12	n.d.	29.08	n.d.	n.d.		
	cD10	+	-	+	3030.27	22.34	n.d.	n.d.	n.d.	+	-
					2886.34	21.93	n.d.	n.d.	n.d.		

					3154.58	30.16	n.d.	n.d.	n.d.		
					1454.53	7.75	n.d.	n.d.	n.d.		
	cD11	+	-	+	1433.26	8.08	n.d.	n.d.	n.d.	-	-
					1500.28	7.53	n.d.	n.d.	n.d.		
					2054.78	7.33	345.22	n.d.	4.89		
	cD20	+	-	+	2163.31	7.58	336.83	n.d.	5.03	+	-
					2247.06	8.01	371.03	n.d.	5.13		
					1103.32	6.66	n.d.	n.d.	n.d.		
	dD31	+	+	-	926.58	7.35	n.d.	n.d.	n.d.	-	-
					1274.31	9.14	n.d.	n.d.	n.d.		
					578.78	7.77	n.d.	20.49	16.09		
	dD32	+	+	-	606.35	8.181	n.d.	17.71	11.29	+	-
					527.82	7.46	n.d.	25.80	20.15		
					751.29	6.88	103.56	200.42	55.74		
	dD62	+	-	+	696.66	5.23	91.84	176.85	60.13	+	-
					707.57	7.12	114.60	195.44	61.29		
					666.66	1.70	243.26	n.d.	n.d.		
	dD64	+	+	-	707.13	2.15	258.48	n.d.	n.d.	-	+
					612.59	1.49	271.31	n.d.	n.d.		
					1702.31	0	2415.03	n.d.	n.d.		
	dD74	+	-	-	1556.65	n.d.	2228.64	n.d.	n.d.	-	-
					1627.03	0	2310.48	n.d.	n.d.		
**Extretory system diseases**					2140.36	4.25	57.45	n.d.	20.51		
	cD5	+	-	-	2372.70	4.72	53.34	n.d.	25.16	+	
					2253.14	3.99	49.81	n.d.	18.84		
					3163.30	2.75	n.d.	n.d.	n.d.		
	cD7	+	+	+	2935.12	3.08	n.d.	n.d.	n.d.	-	-
					3044.32	2.31	n.d.	n.d.	n.d.		
					2020.83	21.38	121.42	n.d.	27.46		
	cD8	+	-	+	1963.31	20.04	117.94	n.d.	20.36	+	+

					2105.55	18.54	110.66	n.d.	31.06		
					1883.25	8.16	n.d.	n.d.	n.d.		
	cD9	+	+	-	2012.10	7.99	n.d.	n.d.	n.d.	+	+
					1917.72	7.42	n.d.	n.d.	n.d.		
					4743.29	3.60	n.d.	51.14	n.d.		
	cD17	+	-	+	5011.26	5.13	n.d.	42.56	n.d.	-	-
					4628.11	3.02	n.d.	60.61	n.d.		
					2933.41	5.92	85.14	n.d.	70.18		
	cD19	+	-	+	3054.51	6.85	82.29	n.d.	73.85	+	
					2874.61	6.25	71.63	n.d.	66.03		
					811.71	2.37	201.85	38.42	74.21		
	cD27	+	+	+	784.84	2.43	175.04	32.23	80.13	+	+
					802.33	1.96	188.96	41.10	70.15		
					6356.6	10.98	n.d.	0	0		
	cD28	-	-	+	6817.31	7.54	n.d.	0	0	-	-
					6528.77	8.64	n.d.	0	0		
					2023.64	n.d.	n.d.	0	40.52		
	dD38	+	+	-	1803.01	n.d.	n.d.	0	47.08	+	+
					2140.36	n.d.	n.d.	0	39.59		
					3155.82	n.d.	n.d.	39.62	57.43		
	dD44	+	+	-	2981.79	n.d.	n.d.	38.58	68.12	-	+
					3312.36	n.d.	n.d.	41.26	60.26		
					266.66	1.76	451.67	82.40	n.d.		
	dD61	+	-	+	311.05	1.93	463.23	85.41	n.d.	+	-
					287.12	2.15	430.16	90.88	n.d.		
					936.35	1.25	92.40	n.d.	n.d.		
	dD73	+	-	+	1025.91	1.77	88.17	n.d.	n.d.	-	-
					995.741	1.51	101.22	n.d.	n.d.		
**Digestive system diseases**					2684.82	8.83	42.36	n.d.	41.09		
	cD6	+	+	+	2735.29	7.91	36.52	n.d.	47.29	+	-

					2431.64	10.15	45.72	n.d.	36.55		
	cD18	+	-	+	3925.11	1.54	n.d.	n.d.	37.12	+	-
					4024.20	1.73	n.d.	n.d.	45.52		
					4102.78	2.03	n.d.	n.d.	41.03		
	cD23	+	-	+	4372.68	7.01	n.d.	24.64	23.11	+	-
					4812.37	7.52	n.d.	29.04	19.82		
					4578.62	6.89	n.d.	31.15	27.44		
	dD37	+	+	-	342.42	n.d.	n.d.	n.d.	n.d.	-	-
					298.53	n.d.	n.d.	n.d.	n.d.		
					303.58	n.d.	n.d.	n.d.	n.d.		
	dD63	+	-	+	452.18	0.47	183.07	n.d.	11.27	-	+
					392.15	0.56	202.59	n.d.	15.40		
					436.66	0.59	189.60	n.d.	16.82		
	dD66	+	-	-	548.33	2.36	30.17	99.15	n.d.	-	-
					602.11	1.58	28.22	101.45	n.d.		
					589.99	1.75	24.87	118.26	n.d.		
	dD70	+	+	-	254.92	n.d.	n.d.	n.d.	n.d.	-	-
					315.80	n.d.	n.d.	n.d.	n.d.		
					293.33	n.d.	n.d.	n.d.	n.d.		
	dD71	+	-	+	5036.94	4.02	n.d.	20.18	n.d.	-	-
					4729.16	2.88	n.d.	17.43	n.d.		
					4869.95	3.22	n.d.	22.58	n.d.		
	dD72	-	-	+	1596.95	3.48	296.17	56.10	n.d.	+	-
					1325.08	3.03	303.47	55.14	n.d.		
					1411.63	2.98	288.74	50.69	n.d.		
	dD75	+	-	+	973.32	n.d.	100.00	n.d.	n.d.	-	-
					1027.83	n.d.	101.44	n.d.	n.d.		
					958.04	n.d.	83.12	n.d.	n.d.		
**Skeletal system diseases**	dD41	+	+	+	612.34	n.d.	141.29	n.d.	n.d.	+	-
					556.66	n.d.	156.92	n.d.	n.d.		

				645.80	n.d.	123.68	n.d.	n.d.		
				606.81	n.d.	40.19	n.d.	n.d.		
dD65	+	-	+	612.32	n.d.	47.52	n.d.	n.d.	-	-
				549.99	n.d.	38.97	n.d.	n.d.		
				1125.89	n.d.	33.18	n.d.	15.45		
dD67	+	-	+	1079.99	n.d.	36.35	n.d.	18.22	-	-
				993.15	n.d.	42.16	n.d.	16.31		
				531.42	36.55	388.27	n.d.	n.d.		
dD76	+	-	+	566.66	42.14	391.77	n.d.	n.d.	-	-
				505.28	41.52	404.52	n.d.	n.d.		

c – cat, d – dog, D – diet, n.d. – not detected, *Fv – Fusarium verticillioides, Fp – Fusarium proliferatum.*
